# Uniparental silencing of 5S rRNA genes in plant allopolyploids – insights from *Cardamine* (Brassicaceae)

**DOI:** 10.1111/tpj.16850

**Published:** 2024-06-05

**Authors:** Terezie Mandáková, Alice Krumpolcová, Roman Matyášek, Roman Volkov, Martin A. Lysak, Ales Kovařík

**Affiliations:** ^1^ Central European Institute of Technology (CEITEC) Masaryk University 625 00 Brno Czech Republic; ^2^ Department of Experimental Biology, Faculty of Science Masaryk University 611 37 Brno Czech Republic; ^3^ Department of Molecular Epigenetics, Institute of Biophysics Academy of Sciences of the Czech Republic 612 00 Brno Czech Republic; ^4^ Department of Molecular Genetics and Biotechnology Yuriy Fedkovych Chernivtsi National University 58012 Chernivtsi Ukraine; ^5^ Faculty of Science, National Centre for Biomolecular Research Masaryk University 625 00 Brno Czech Republic

**Keywords:** rRNA genes, 5S rDNA, 35S rDNA, *Cardamine*, polyploidy, gene silencing, chromosome evolution

## Abstract

While the phenomenon of uniparental silencing of 35S rDNA in interspecific hybrids and allopolyploids is well documented, there is a notable absence of information regarding whether such silencing extends to the 5S RNA component of ribosomes. To address this gap in knowledge, we analyzed the 5S and 35S rDNA expression in *Cardamine* (Brassicaceae) allopolyploids, namely *C.* × *insueta* (2*n* = 3*x* = 24, genome composition RRA), *C. flexuosa* (2*n* = 4*x* = 32, AAHH), and *C. scutata* (2*n* = 4*x* = 32, PPAA) which share a common diploid ancestor (AA). We employed high‐throughput sequencing of transcriptomes and genomes and phylogenetic analyses of 5S rRNA variants. The genomic organization of rDNA was further scrutinized through clustering and fluorescence *in situ* hybridization. In the *C.* × *insueta* allotriploid, we observed uniparental dominant expression of 5S and 35S rDNA loci. In the *C. flexuosa* and *C. scutata* allotetraploids, the expression pattern differed, with the 35S rDNA being expressed from the A subgenome, whereas the 5S rDNA was expressed from the partner subgenome. Both *C. flexuosa* and *C. scutata* but not *C.* × *insueta* showed copy and locus number changes. We conclude that in stabilized allopolyploids, transcription of ribosomal RNA components occurs from different subgenomes. This phenomenon appears to result in the formation of chimeric ribosomes comprising rRNA molecules derived from distinct parental origins. We speculate that the interplay of epigenetic silencing and rDNA rearrangements introduces an additional layer of variation in multimolecule ribosomal complexes, potentially contributing to the evolutionary success of allopolyploids.

## Significance Statement

A scheme showing evolutionary relationships among *Cardamine* allopolyploids and their diploid progenitors. Arrows depict the direction of interspecific hybridization. Drawings after the species name indicate ribosomes and their ribosomal RNA (rRNA) composition. Individual rRNA molecules are indicated by colored lines. The rRNA types (boxed, upper right) showed uniparental inheritance in the *C.* × *insueta* allotriploid and a biparental inheritance in *C. flexuosa* and *C. scutata*.

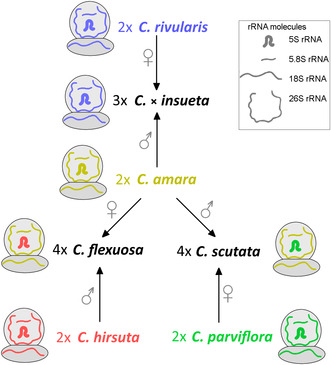

Ribosome biogenesis is a critical process for eukaryotic cells, demanding the orchestrated synthesis of protein and ribosomal RNA (rRNA) components. In this study, we demonstrate that plant allopolyploidy (the conditions where plants possess multiple sets of chromosomes from different species) is linked with the formation of chimeric ribosomes comprising rRNA molecules of distinct parental origins. Variation in multi‐molecular ribosomal complexes may contribute to the evolutionary success observed in allopolyploids.

## INTRODUCTION

Ribosome biogenesis is a critical process for eukaryotic cells, demanding the orchestrated synthesis of protein and rRNA components, both highly regulated. Eukaryotic ribosomes consist of four RNA molecules (5S, 5.8S, 18S, and 26S/28S) encoded by the polycistronic 35S (45S in animals) rDNA (18S–5.8S–26S/28S rRNA) and 5S rDNA. Among these, 5S ribosomal RNA (5S rRNA) is a small but integral constituent of the large ribosomal subunit. Though not definitely proven, 5S rRNA is believed to mediate allosteric interactions between ribosomal functional centers (Gongadze, 2011; Kouvela et al., 2007) and significantly contribute to efficient and accurate ribosome assembly (Huang et al., 2020). On the ribosome, 5S rRNA establishes multiple contacts with structural elements linked to the peptidyl transferase center, elongation factor binding region, and mRNA decoding site (Sáez‐Vásquez & Delseny, 2019; Szymanski et al., 2003). Its intensive involvement in intraribosomal interactions imposes stringent structural and functional constraints on evolutionary changes, rendering 5S rRNA one of the most conserved ribosomal components in terms of sequence, secondary structure, and three‐dimensional layout (Sun & Caetano‐Anolles, 2009; Szymanski et al., 1999). In contrast, noncoding intergenic spacers located between neighboring genes exhibit rapid evolution, often displaying strong phylogenetic signals (Alexandrov et al., 2021; Cardoni et al., 2021; Röser et al., 2001; Sergeeva et al., 2017; Tynkevich et al., 2022; Volkov et al., 2001).

Regulation of 5S rDNA transcription relies on an internal tripartite RNA polymerase III promoter and 5ʹ‐prime upstream sequences (Hemleben & Werts, 1988; Vaillant et al., 2007). Besides specific factors, general epigenetic regulators, including DNA and histone modifications, contribute to the regulation of 5S transcription in plants. Nucleolar dominance, an epigenetic phenomenon observed in numerous interspecific hybrids and allopolyploids across both plant and animal kingdoms, entails the exclusive transcription of 35S rRNA genes inherited from one evolutionary progenitor (Borowska‐Zuchowska et al., 2023; Pikaard et al., 2023). Molecular mechanisms underlying nucleolar dominance often (Borowska‐Zuchowska et al., 2020; Chen & Pikaard, 1997; Guo & Han, 2014; Houchins et al., 1997; Neves et al., 1995) but not always (Schubert & Kunzel, 1990) involve DNA methylation and repressive histone marks (Earley et al., 2006). In stark contrast to the well‐studied 35S rRNA genes, little is known about the expression status of parental 5S rDNA in allopolyploids, primarily due to challenges in discriminating between homologous transcripts. However, locus‐specific silencing has been demonstrated in the diploid *Arabidopsis thaliana* (Cloix et al., 2000; Simon et al., 2018). A major locus on chromosome 5 exhibits high expression, whereas minor loci on chromosomes 2 and 3 are silenced or expressed less intensively. This observation suggests that similar to the 35S rDNA loci, a hierarchical pattern of expression also exists for the 5S rDNA loci. Naturally occurring allopolyploids provide ideal systems for investigating the genetic and epigenetic consequences of hybridization and genome doubling on the fate of paternally and maternally inherited 5S rRNA genes.

**Figure 1:**
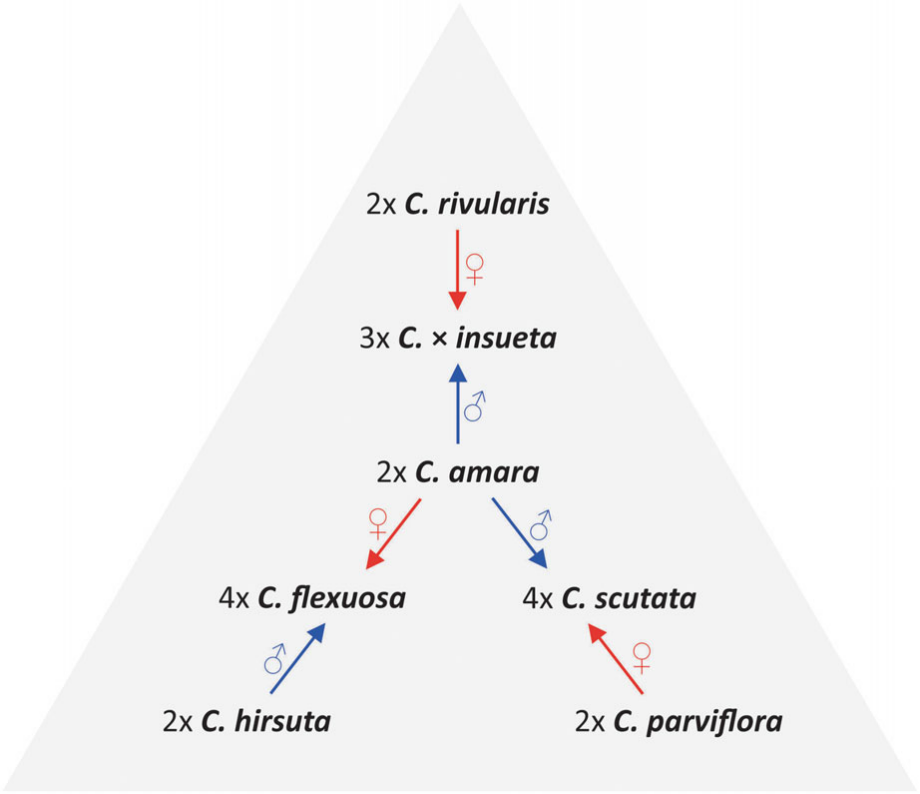
Evolutionary relationships among *Cardamine* allopolyploids and their diploid progenitors. Arrows depict the direction of interspecific hybridization. Recurrent hybridization event involved *C. amara* as either a paternal (*C*. × *insueta*, *C. scutata*) or a maternal (*C. flexuosa*) genome donor (Lihova et al., 2006; Mandáková et al., 2013; Zozomová‐Lihová et al., 2014).

The genus *Cardamine* L. (bittercress) stands as one of the largest genera of the family Brassicaceae, encompassing over 200 species distributed across all continents except Antarctica (Lihova & Marhold, 2006). Frequent instances of spontaneous interspecific hybridization have been documented among various *Cardamine* species (e.g., Mandáková et al., 2013, 2014, 2019). This genus exhibits a notable prevalence of polyploidy (Lihova & Marhold, 2006). Consequently, both hybridization and polyploidy emerge as pivotal mechanisms propelling speciation within the genus *Cardamine* (Mandáková et al., 2019). While postpolyploid evolution typically involves genome diploidization, including substantial chromosome rearrangements and descending dysploidy (Mandáková & Lysak, 2018), *Cardamine* polyploids demonstrate remarkable genome stability. With the exception of *C. cordifolia* (Mandáková et al., 2016) and *C. pratensis* (Mandáková et al., 2013), instances of postpolyploid descending dysploidy have not been reported in other *Cardamine* species, and their karyotypes do not manifest significant major rearrangements. In contrast to the rest of the genome, the 35S rDNA loci undergo rearrangements associated with locus and copy number changes in recently formed *C. schulzii* (Zozomova‐Lihova et al., 2014) and more ancient *C. flexuosa* (Mandáková et al., 2014) allopolyploids. Evidence for rapid homogenization of 35S rDNA in *Cardamine* arises from cloning experiments (Franzke & Mummenhoff, 1999; Zozomová‐Lihová et al., 2014) and cytogenetic observations (Mandáková et al., 2014; Zozomová‐Lihová et al., 2014). In this study, our objectives were to elucidate the presence, if any, of 5S rDNA dominance – a counterpart to nucleolar dominance – in plant allopolyploids. Additionally, we explored the coordination of 5S rDNA expression with that of 35S rDNA, investigating whether both transcripts originated from the same or different subgenomes. Last, we assessed the integrity of parental rDNA loci in allopolyploids with varying origins, ages, and populations. To address these questions, we scrutinized rRNA variants in sequenced transcriptomes and their corresponding genomes. The experimental model system involved three distinct *Cardamine* allopolyploids, namely *C*. × *insueta* (2*n* = 3*x* = 24, genome composition RRA), *C. flexuosa* (2*n* = 4*x* = 32, AAHH), and *C. scutata* (2*n* = 4*x* = 32, PPAA) (Figure 1). Each allopolyploid incorporated a shared parental genome derived from *C. amara* (2*n* = 16, AA), coupled with diverse partner genomes sourced from *C. rivularis* (RR), *C. hirsuta* (HH), and *C. parviflora* (PP) (all 2*n* = 2*x* = 16) (Table 1).

**Table 1:** Cytogenetic characteristics of rDNA loci in *Cardamine* analyzed by FISH

Species	Ploidy level	Genomic compositon	Maternal parent	Paternal parent	5S rDNA sites per 2C^a^	35S rDNA sites per 2C^a^
*C. amara*	2*x*	AA	‐	‐	2	4
*C. rivularis*	2*x*	RR	‐	‐	4	10
*C. hirsuta*	2*x*	HH	‐	‐	2	6
*C. parviflora*	2*x*	PP	‐	‐	2	2
*C. × insueta*	3*x*	RRA	*C. rivularis*	*C. amara*	3–4 (1 A + 2–3 R)	10–12 (2 A + 8–10 R)
*C. flexuosa*	4*x*	AAHH	*C. amara*	*C. hirsuta*	2 (2 A + 0 H)	4 (4 A + 0 H)
*C. scutata*	4*x*	PPAA	*C. amara*	*C. parviflora*	6 (0 A + 6 P)	2 (2 A + 0 P)

FISH, fluorescent *in situ* hybridization.

^a^ The source data are in Figure 6; Figures S3 and S4. The number of rDNA sites in each subgenome is in brackets.

## RESULTS

### *Cardamine* diploids exhibit substantial polymorphisms in the 5S rRNA coding regions

**Figure 2:**
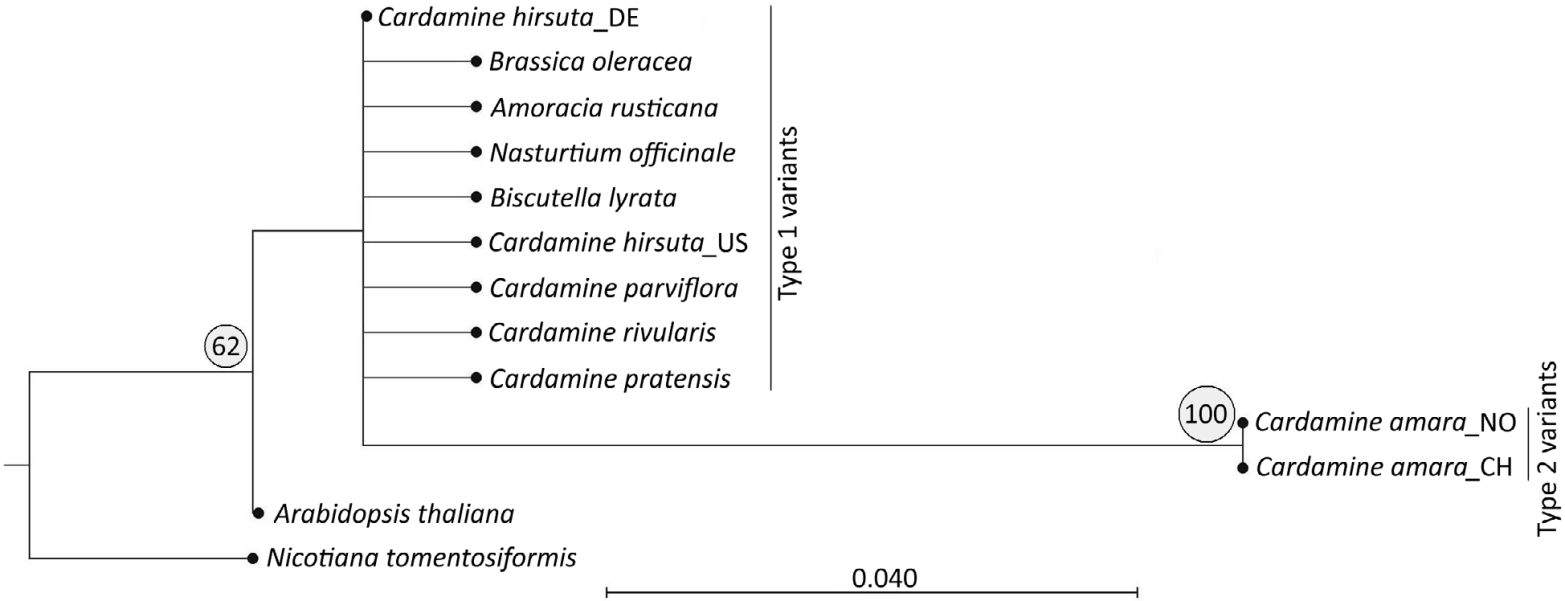
A neighbor‐joining dendrogram showing phylogenetic analysis of 5S rRNA coding sequences in Brassicaceae. Note that the *Cardamine amara* 5S genes from two populations are indistinguishable and form a distinct clade. The *Nicotiana tomentosiformis* 5S sequence (GenBank, AJ131168.1) was used as an outgroup. A bootstrap support value is circled.

**Figure 3:**
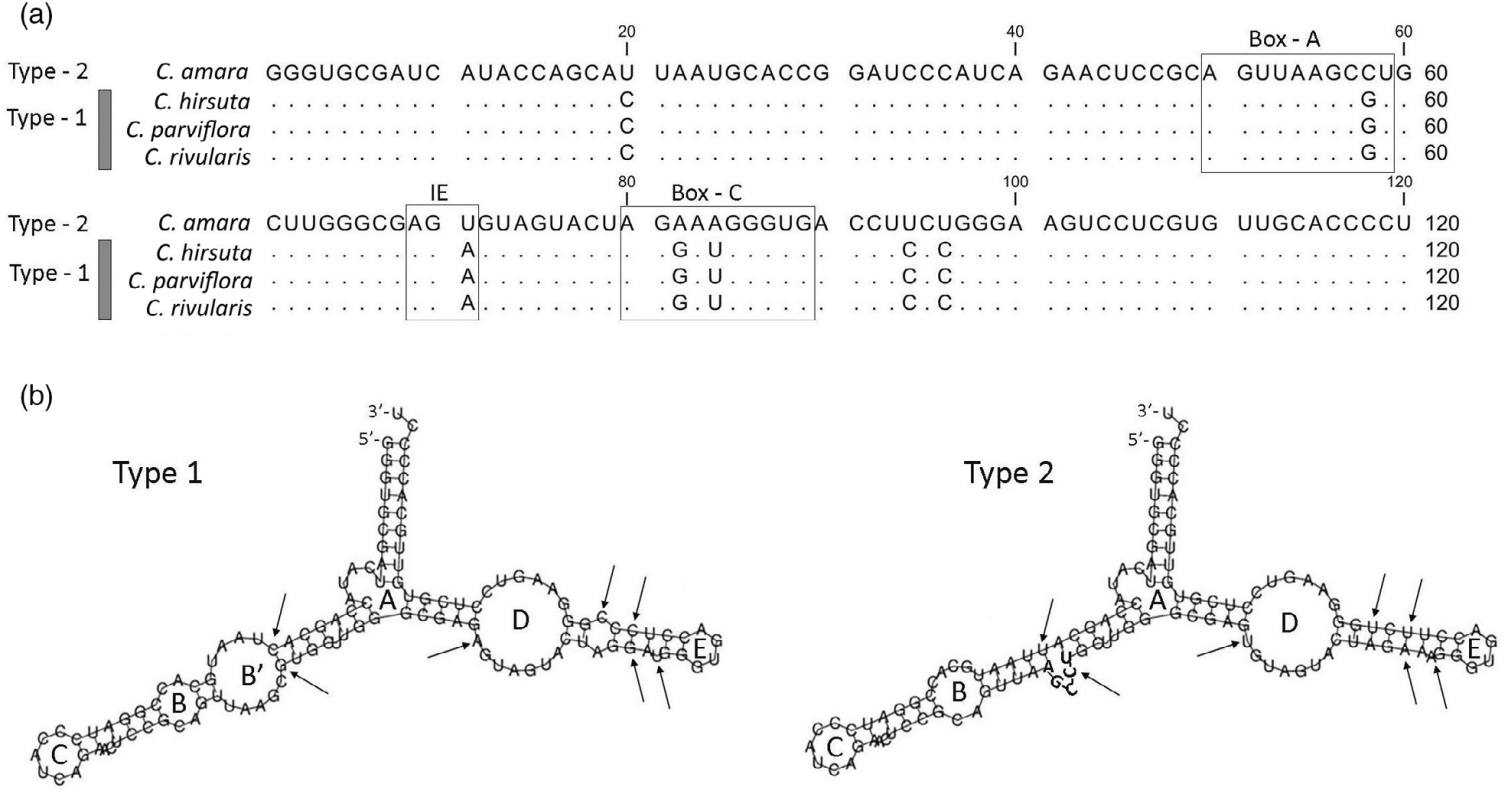
Sequences and secondary structures of most common 5S rRNA types in *Cardamine* diploids. (a) Alignment of 120 nt‐long rRNA molecules. Dots are according to the *C. amara* sequence. The RNA polymerase III promoter region is composed of boxes A and box C and internal element (IE) is boxed. Note that substantial variation in all three promoter elements. (b) Predicted secondary structures of 5S rRNA variants. The free energy values reflecting the thermal stability of type 1 and type 2 variants were −38.6 and −37.4 kcal mol^−1^, respectively. Positions of polymorphic sites are indicated by arrows. The names of the loops follow the nomenclature of Barciszewska et al. (1994).

The 5S genic region, ~120 bp in length, facilitates the reconstruction of near‐complete 5S rRNA sequences from short Illumina reads. Our analysis encompassed multiple RNA‐seq libraries from four diploid species and their derived allopolyploids (Table 1; Table S1). In diploids, the 5S rRNA gene diversity was generally low, ranging from 0.158 to 0.775 (Table 2; Table S2). For instance, in *C. amara* and *C. hirsuta*, 94–96% of 5S rRNA transcripts were dominated by a single variant. *C. parviflora* exhibited three relatively abundant variants, indicating slightly higher diversity of 5S rRNA pools than the other two species. On a phylogeny tree (Figure S1) constructed from hundreds of 5S read sequences from diploid species, all sequences from *C. hirsuta*, *C. rivularis*, and *C. parviflora* clustered as one clade, denoted as type 1 variant. In contrast, reads from *C. amara* formed a distinct clade called type 2 variant. Phylogenetic analysis, including several *Cardamine* and non‐*Cardamine* species, revealed that the type 1 variant grouped with 5S rRNA genes of other Brassicaceae species, such as *Arabidopsis thaliana*, *Brassica oleracea*, *Biscutella laevigata*, whereas the type 2 variant was unique to *C. amara* (Figure 2). Alignment of major 5S rRNA types (Figure 3a) from diploid species revealed seven substitutions (94% identity) between type 1 and type 2. These polymorphic sites defined a species‐specific pattern, and corresponding single nucleotide polymorphisms (SNPs) were utilized for discrimination between 5S homologous transcripts in allopolyploids (further below). The polymorphic sites were evenly distributed along the 120 nt‐long genic regions, encompassing the RNA polymerase III promoter composed of Box A (one polymorphic site), Box C (two sites), and internal element (one site). Conversely, the TATA box at −28 and a T‐rich terminator beyond +120 showed no polymorphisms (Figure S2). SNP analysis revealed essentially the same variants across different populations of diploid species (Table S3). At the DNA level, the 5S rDNA diversity was higher than in the transcriptomes, ranging from 0.748 to 0.920. Yet, a single dominant type constituted 42–53% of total 5S rDNA (Table 2; Table S2), aligning with a major 5S RNA transcript.

**Table 2:** Diversity of 5S rRNA and 5S rDNA in *Cardamine* diploids

Species	Sample	Number of reads	Number of variants	Diversity	Major 5S variant^a^		
					Count	(%)^b^	(%)^c^
*C. amara*	Transcriptomic	149	20	0.296	125	83.9	95.0
	Genomic	256	83	0.842	100	39.1	47.6
*C. hirsuta*	Transcriptomic	667	37	0.158	612	91.8	96.4
	Genomic	208	97	0.92	57	27.4	41.9
*C. parviflora*	Transcriptomic	262	40	0.775	113	43.1	49.6
	Genomic	613	92	0.748	292	47.6	53.4

^a^ Frequencies of other less abundant variants are given in Table S2. Diversity (haplotype) was calculated according to Nei (1987, equations 8.4 and 8.12), but replacing 2*n* by *n* in a formula used by a DNaSP5 program.

^b^ All variants.

^c^ After exclusion of singletons.

To assess the potential impact of mutations on secondary structures, and consequently functionality, we employed the RNA‐fold program to model the secondary structures of 5S rRNA molecules (Figure 3b). A typical Y‐shape structure was conserved between both type 1 and type 2 variants. The thermal stability of both structures was comparable. In both structures, four of seven polymorphic sites were located in stems; three were in unpaired regions. Subtle structural differences between type 1 and type 2 variants included: (i) Additional loop (B′) formed in type 1. Its 5′ closing CG pair involved C at +20 and G at +58. Both positions were located within the polymorphic sites. (ii) A three‐base bulge in a stem between loops A and B in type 2. (iii) A G:A mismatch close to the D loop in type 1. In conclusion, it appears that the two 5S rRNA variants identified in *Cardamine* diploids differed significantly by their primary sequence but exhibited minimal distinctions in their secondary structures.

### Expression of 5S and 35S rDNA in hybrids and allopolyploids

**Figure 4:**
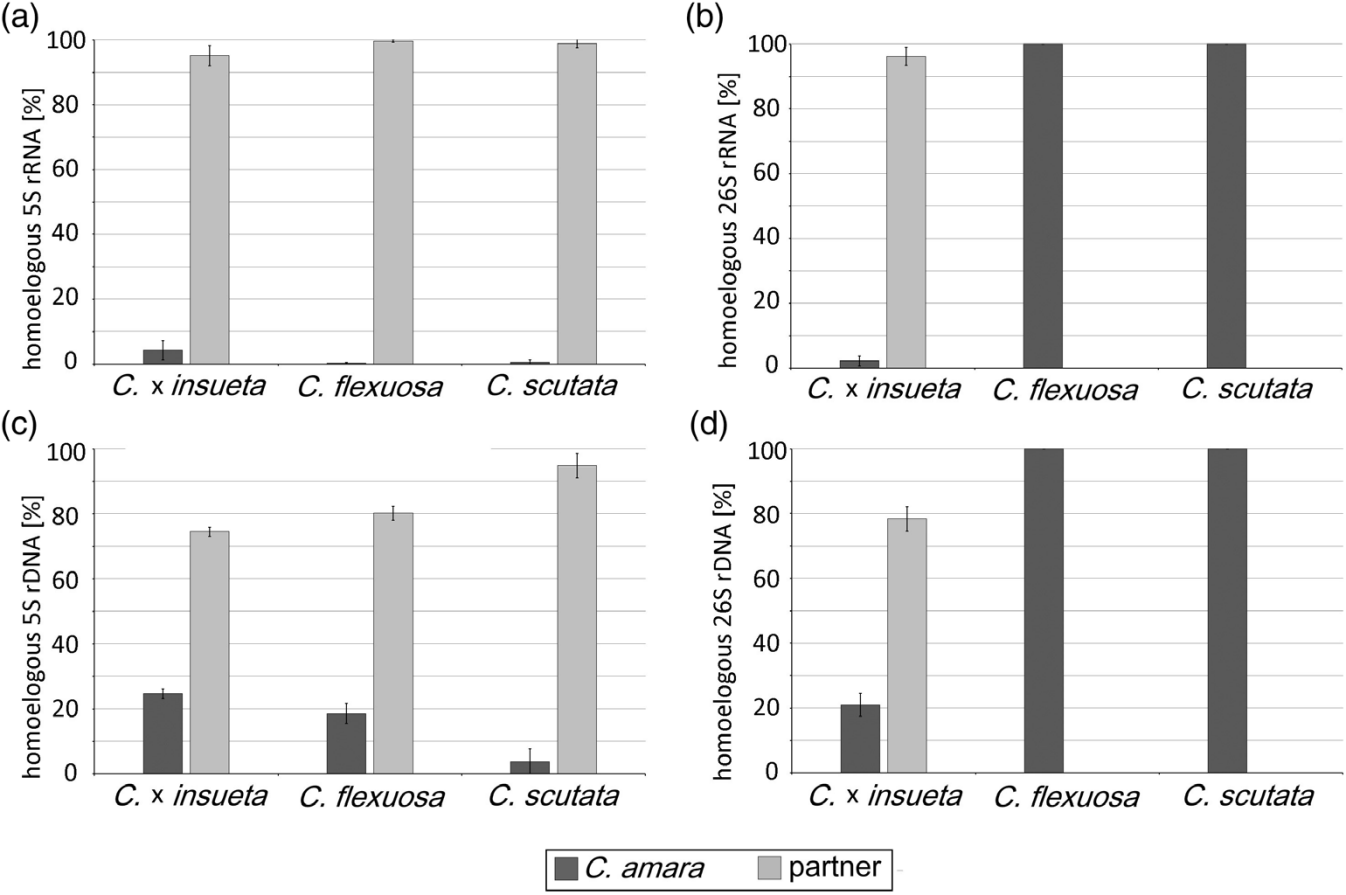
Transcription and genomic analysis of 5S and 35S rDNA in *Cardamine* hybrids and allopolyploids. (a, b) Proportions of homologous 5S rRNA (a) and 26S rRNA (b) transcripts were calculated from the single nucleotide polymorphism analyses of transcriptomes (Tables [Link], [Link], sheet 1). Values obtained from different accessions were averaged and graphically expressed. Variation between the accessions (four cDNA libraries of each *C*. × *insueta* and *C. flexuosa*) was negligible (c, d). Genomic proportions of homologous 5S rDNAs (c) and 26S rDNAs (d) calculated from the single nucleotide polymorphism analyses of genomic reads (Tables [Link], [Link], sheet 2).

The sequence divergence within the coding regions of rDNA units provided the basis for analyzing the origin of 5S rRNA transcripts in *Cardamine* allopolyploids. Seven polymorphic sites distinguishing type 1 and type 2 variants (Figure 3a) were employed in SNP analysis of transcriptomes from four accessions of each *C*. × *insueta* (Table S4) and *C. flexuosa* (Table S5), and a single accession of *C. scutata* (Table S6). In all accessions, SNPs specific to type 1 variant (inherited from *C. hirsuta*, *C. rivularis*, and *C. parviflora*, respectively) dominated the 5S rRNA pools (Figure 4a). Similarly, the reconstructed 5S rRNA types from random‐primed total RNA libraries exhibited a substantial bias toward the type 1 variant (Table 3). To assess the expression levels of homologous 26S rRNA genes (a marker indicative of dominance), we conducted SNP analysis on transcriptomes. Approximately, 10–33 polymorphic sites (pairwise comparison) were identified in the 26S rRNA genes among diploid species (Table S7). In *C*. × *insueta*, SNPs were predominantly biased toward the *C. rivularis* parent (Figure 4b; Table 3; Table S4), while those from *C. amara* were nearly absent. Conversely, both *C. flexuosa* (Table S5) and *C. scutata* (Table S6) exhibited SNPs specific to the *C. amara* sequence only (Figure 4b), while those of the other genomes were rare (Table 3).

**Table 3:** The inheritance of parental 5S rDNA variants in *Cardamine* allopolyploids

Species	Sample	Accession	Total reads	*C. amara* subgenome		Partner subgenome	
				Reads	(%)	Reads	(%)
*C. insueta*	Transcriptomic	PRJDB9426^a^	327	8	2.4	319	97.6
	Genomic	SUB14263739	109	21	19.3	88	80.7
*C. flexuosa*	Transcriptomic	SRR26392759	183	0	0	183	100
	Genomic	SRR10230721	519	66	13	444	87
*C. scutata*	Transcriptomic	SRR26391928	156	0	0	156	100
	Genomic	SRR27971144	935	15	2	920	98

^a^ Pooled reads from archives DRR216619, DRR216617, DRR216621, and DRR216624.

### Genome proportions and rDNA copy number variation

The interpretation of transcriptomic profiles in allopolyploid genomes relies on the intactness of loci and the retention of progenitor genes. Consequently, we carried out an analysis of genomic reads to determine the genome proportion of homologous 5S and 26S rDNAs in *C. × insueta*, *C. flexuosa*, and *C. scutata*. Both parental 5S rDNA variants were identified in allopolyploids (Figure 4c). In contrast, the genome proportion of 26S rDNA exhibited a pronounced skew toward the A subgenome homolog (Figure 4d), particularly evident in *C. flexuosa* and *C. scutata*. Subsequently, we calculated the copy number of homologous rDNA in allopolyploids (Table S8). The 5S rDNA copy number ranged from 114 to 543 in the A genome and from 580 to 4930 in the partner subgenome. Similarly, the 26S copy number ranged from 219 to 1244 in the A genome and from 12 to 817 in the partner subgenomes.

### Genomic organization of 5S rDNA

**Figure 5:**
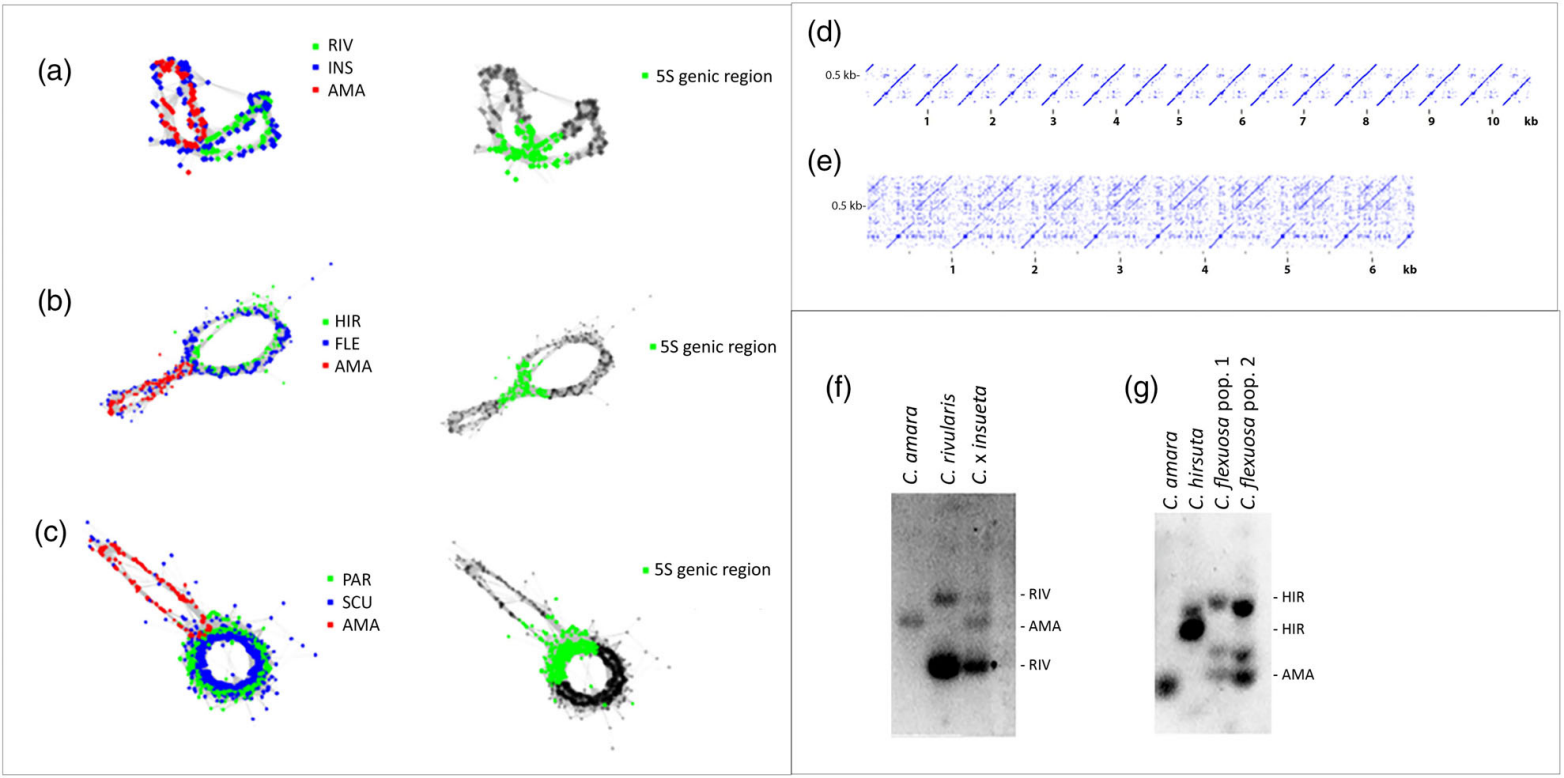
The inheritance of 5S rDNA variants in *Cardamine* allopolyploids. (a–c) Comparative cluster graph analysis of 5S rDNA genomic reads. Similarity between reads (dots) is indicated by interconnected lines. The progenitor 5S reads were labeled in red (*C. amara* – AMA) and green (*C. hirsuta* – HIR, *C. rivularis* – RIV, and *C. parviflora* – PAR) dots. Reads derived from allopolyploids are in blue dots. Overlaps between blue and other colors indicate retention of a progenitor variant in allopolyploid. (d, e) Dotplot analysis of PacBio pseudomolecules carrying short (d) and long (e) variants of IGS. The *Y*‐ and *X*‐axes represent unit monomers and pseudomolecules, respectively. (f, g) Southern blot hybridization analysis of 5S rDNA units in *C*. × *insueta* and *C. flexuosa* and their progenitors. The MboI restriction fragments were hybridized against the 5S rDNA probe. Note, additivity of restriction fragments in *C*. × *insueta*.

It is well established that the 5S intergenic spacers represent fast‐evolving region of rDNA units. Therefore, we investigated whether the intergenic spacers remained intact or underwent molecular evolution in allopolyploids. To analyze IGS sequences at the genomic level, we employed clustering analysis, a convenient tool for identifying 5S families and their parental origin (Garcia et al., 2020). The projections of cluster graphs are depicted in Figure 5(a–c). In these graphs, each loop represents a distinct 5S family bearing a unique IGS sequence, with the central part composed of reads from shared genic sequences. The blue dots represent reads from allopolyploid species, and their overlap with reads from parental species (red and green) suggests the absence of major rearrangements. The allopolyploid‐specific reads were relatively distributed between both loops in Figure 5(a,b). In *C. scutata* (Figure 5c, blue dots), the overlap with reads from *C. parviflora* only is consistent with the high and low proportion of 5S rDNA in the P subgenome and A subgenome, respectively (Figure 4). In order to determine a higher‐order structure of 5S rDNA in *C. flexuosa*, we analyzed long PacBio reads. The dot plot analysis of 22 reads revealed two types of repeats (Figure 5d,e). Twenty (90%) reads contained tandems with a repeat length of ca. 650 bp. These units were highly similar to *C. hirsuta* (type 1 variant) (Figure 5d). Two (10%) reads had longer (c. 720) monomers containing 5S genic regions similar to *C. amara* (type 2 variant) (Figure 5e). Southern blot hybridization revealed multiple bands in both *C*. × *insueta* and *C. flexuosa* (Figure 5f,g). Full additivity of parental bands was observed in *C*. × *insueta*, whereas *C. flexuosa* exhibited some deviation from additivity, with a strong band located between those of *C. amara* and *C. hirsuta* visualized.

### Chromosome positioning of rDNA

**Figure 6:**
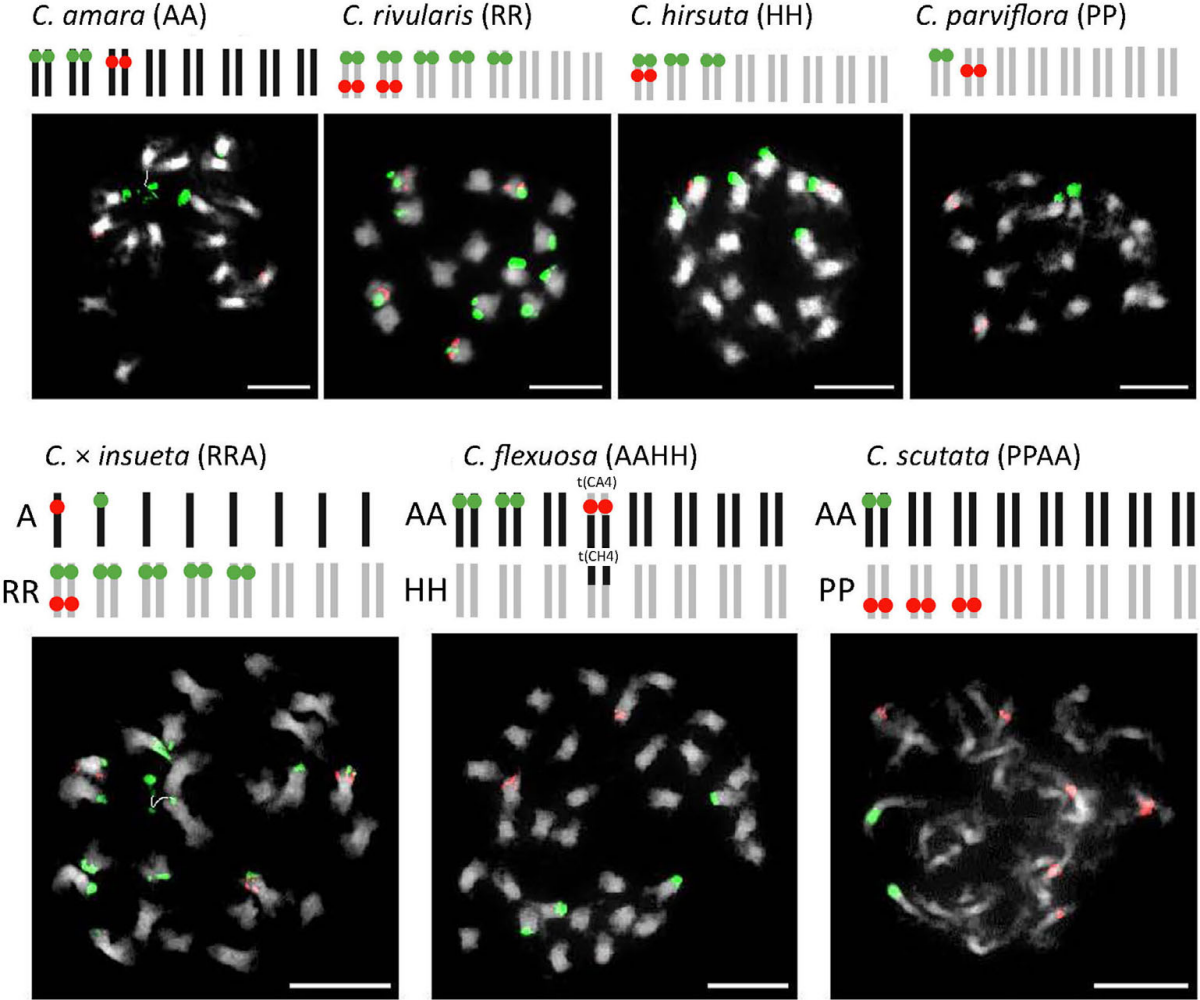
Fluorescent *in situ* hybridization analysis of rDNA loci in *Cardamine* diploids and allopolyploids. Metaphase chromosomes were subjected to hybridization using 5S (in red) and 18S (in green) rDNA probes. Chromosomes were counterstained with 4′,6‐diamidino‐2‐phenylindole. Scale bars: 10 μm. Schematic representations illustrate the distribution of 5S (in red) and 18S (in green) rDNA loci in diploid *C. amara* (AA), *C. rivularis* (RR), *C. hirsuta* (HH), and *C. parviflora* (PP) (all 2*n* = 16) as well as allopolyploid *C*. × insueta (2*n* = 3*x* = 24, RRA), *C. flexuosa* (2*n* = 4*x* = 32, AAHH), and *C. scutata* (2*n* = 4*x* = 32, PPAA). Chromosome ideograms are colored according to their origin. Note a prominent intergenomic translocation between homoelogous chromosomes 4 of *C. flexuosa*.

To determine the number and position of rDNA loci on chromosomes, we conducted fluorescent *in situ* hybridization (FISH) analysis targeting 5S and 18S rDNA loci. The parental diploid species exhibit distinct rDNA patterns as follows: *C. amara* (AA) possesses two chromosome pairs harboring 18S rDNA and one chromosome pair bearing 5S rDNA. *C. hirsuta* (HH) displays three chromosome pairs with 18S rDNA, with one of them additionally bearing 5S rDNA. *C. parviflora* (PP) is characterized by one chromosome pair carrying 18S rDNA and another pair bearing 5S rDNA. *C. rivularis* (RR) features five chromosome pairs with 18S rDNA, and two of them also harbor 5S rDNA. Notably, all 18S rDNA loci were identified in chromosome termini, while the 5S rDNA was observed in interstitial position (Figure 6). In the analysis of 20 individuals of *C*. × *insueta* (RRA), a subtle variability in the count of 5S and 18S sites was observed. The 18S probe exhibited hybridization to 10–12 sites, distributed across 4–5 R chromosome pairs and a single A subgenome chromosome, all situated in subtelomeric positions. The 5S rDNA probe demonstrated hybridization to 3–4 sites, including one on the A subgenome chromosome and 2–3 on the R chromosomes (Figure 6; Figure S3).

In seven analyzed populations of *C. flexuosa* (AAHH), a conserved rDNA pattern was observed. We identified two chromosome pairs containing 18S rDNA and one A/H translocation carrying 5S rDNA. Through the use of chromosome‐specific BAC probes, we determined that the A3 and A7 chromosomes harbor the 18S rDNA, whereas the A4 chromosome, via an intergenomic translocation, acquired the major portion of the upper arm of the H4 chromosome, which bears the 5S rDNA (Mandáková et al., 2014; Figure 6; Figure S4). Thus, the H subgenome seems to have lost both 5S and 18S sites. It remains to be determined whether elimination of all rRNA genes might be a hallmark of the subgenomic dominance reported, for example, in *Festuca* (Poaceae) hybrids (Mahelka et al., 2023). Previous hypotheses suggested that the 5S rDNA served as a fragile site for the intergenomic translocation between two homologous chromosomes A4 and H4, contributing to the postpolyploid chromosome repatterning process in the allotetraploid *C. flexuosa* (Mandáková et al., 2014). Our detailed analysis of pachytene chromosomes revealed the splitting of the 5S locus into closely located loci during this rearrangement (Figure S5).

In *C. scutata* (PPAA), our FISH data revealed a single chromosome pair within the A subgenome bearing terminal 18S rDNA. Conversely, 5S rDNA sites were exclusively identified within the P subgenome, specifically in the pericentromeric region of three chromosome pairs (Figure 6).

In conclusion, the FISH data unveiled that in the tetraploid genomes of *C. flexuosa* and *C. scutata*, the 5S rDNA loci are exclusively situated within the H and P subgenomes, respectively. In the triploid *C*. × *insueta*, the majority of 5S loci is found within the R subgenome. The data collectively indicate a significant elimination of the 5S rDNA loci originating from the A subgenomes in all three allopolyploids. This observation aligns with the expression data, revealing a dominance of the type 1 (non‐A) variant.

## DISCUSSION

### Unusual variation of 5S rDNA coding region in *Cardamine*

The ribosomal 5S RNA gene, typically characterized by high conservation, exhibited surprising diversity in two variants identified in *Cardamine*, differing as much as by 6% along the 120‐nt coding region. Despite this divergence, both variants were completely homogenized, forming hundreds of copies in diploid genomes. Phylogenetic analysis revealed that the type 1 variant is abundantly present in other Brassicaceae genomes, likely representing the ancestral type (Figure 2). In contrast, the type 2 variant appears to be specific for *C. amara*. The mutation spectrum from type 1 to type 2 showed that more than 70% of substitutions were from C to T and from G to A, suggesting that the type 2 variant arose from the ancestral type through processes involving cytosine deamination, drift, and homogenization.

It is noteworthy that a similar mutation trend was observed among the *Arabidopsis thaliana* 5S rDNA loci (Simon et al., 2018), indicating that cytosine deamination processes are common in 5S loci and could be related to the overall high level of 5S rDNA methylation (Fulnecek et al., 1998; Simon et al., 2018). Deamination of methylated Cs leads directly to Ts, resulting in subsequent C > T substitution. It has been proposed that C‐to‐T and G‐to‐A mutations are features of non‐functional pseudogenes since increased A + T content decreases thermal stability of RNA secondary structures (Buckler et al., 1997; Tynkevich et al., 2023; Vizoso et al., 2011). However, the identified type 2 variant is not a pseudogene, as its transcripts account for all 5S rDNA transcription in *C. amara*. Moreover, its secondary structure is nearly identical to that of the type 1 variant, indicating that mutations have not influenced the folding of molecules and can be considered as compensatory (or nearly so). Compensatory mutations refer to a situation where the deleterious effect of base substitution at a given site can be suppressed by a compensatory second‐site substitution (Knies et al., 2008). The secondary structure of 5S rDNA thus appears to evolve under purifying selection. We certainly cannot exclude the effects of mutations on the 5S rRNA tertial structure. For example, the U20C and C59G substitutions apparently affected the stability of the B/B′ loop (Figure 3b) which forms a plant‐specific tertial interaction with the E loop (Joachimiak et al., 1990). In conclusion, both type 1 and type 2 variants of 5S rDNA appear to operate effectively in the ribosomes of diploid cells.

### Unidirectional silencing of 5S rDNA in *Cardamine* allopolyploids

An ancestral diploid genome closely related to the extant *C. amara* was the parental genome of the *C. × insueta* allotriploid and *C. flexuosa* and *C. scutata* allotetraploids. However, the *C. amara*‐specific type 2 variant contributed only marginally (less than 4%) to the total 5S rRNA pools in these allopolyploids, with the majority of 5S transcripts originating from the second parental genome (Figure 4). The low transcription levels cannot be attributed to a reduced number of genes, as the ratio between genomic and transcriptomic proportions of A genome units ranged from 6 to 49 (depending on species). FISH data provide additional support to this supposition. This suggests a significant degree of 5S rDNA silencing in *C. amara* genome in allopolyploids. Silencing appears to be independent of meiosis or age of the allopolyploid, as it was observed in *C*. × *insueta*, a recently formed semisterile allotriploid (<200 years).

While the mechanism of 5S rDNA silencing in allopolyploids remains elusive, it is likely associated with epigenetic modifications, including DNA and histone methylation. Studies in *Arabidopsis* have demonstrated that mutations in epigenetic genes can induce alterations in 5S rDNA (hetero)chromatin and influence the transcription of 5S variants (Layat, Saez‐Vasquez, & Tourmente, 2012; Vaillant et al., 2007). Similar to the phenomenon of nucleolar dominance, the primary trigger for 5S rDNA silencing in *Cardamine* allopolyploids remains unknown. However, it is plausible that silencing is linked to sequence variations in the promoter regions. Notably, the less dominant type 2 variant exhibits extensive mutations in all parts of the tripartite RNA polymerase (Pol III) promoter, including Box‐A, Box C and internal elements. We hypothesize that these mutations may render the Pol III promoter in the type 2 variant less active. Consequently, the relatively weak type 2 promoters (from *C. amara*) might be outcompeted by the strong type 1 promoters (from *C. hirsuta*, *C. parviflora*, and *C. rivularis*) when present in the same nucleus. The promoter strength effect could be further enhanced by a gene dosage effect (Veitia et al., 2013), as the *C. amara* 5S genes are less abundant than the partner genes in *Cardamine* allopolyploids. Genes with weaker promoters are likely more susceptible to variation in transcription factor levels compared to genes with stronger promoters. Supporting this notion, the transcription of 5S rDNA is reliant on the TFIIIA transcription factor, which is present in limited amounts in plant cells, and its levels are known to correlate with the expression activity of 5S rDNA (Layat, Cotterell, et al., 2012). An intriguing avenue for further research would involve determining the strength of Pol III promoters using *in vitro* systems to provide additional support for this hypothesis.

### Both homogeneous and heterogeneous ribosomes in *Cardamine* allopolyploids

Nucleolar dominance, defined as the inactivation of one or more parental 35S rDNA (NOR), serves not only as a potent gene dosage compensating mechanism but also contributes to the uniformity of ribosomes in hybrid and allopolyploid species. This aspect of nucleolar dominance has received limited attention despite evidence from various systems suggesting that 35S rRNA pools are not uniform and may encompass multiple variants (Pontvianne et al., 2010; Sims et al., 2021).

We observed the inactivation of parental 26S and 5S rDNA in all three *Cardamine* allopolyploids investigated. In the *C*. × *insueta* allotriploid, dominance in expression was observed, with the 35S rRNA and 5S rRNA genes inherited from the *C. rivularis* parent, while those from *C. amara* were suppressed. This suggests that the ribosomes in *C*. × *insueta* are homogeneous, resembling the composition of the *C. rivularis* parent. In contrast, in the relatively ancient allotetraploids *C. flexuosa* and *C. scutata*, the 26 rRNA genes were dominantly expressed from the A subgenome, while the 5S rRNA was expressed from the partner (H and P) subgenomes. This indicates that cells in both allopolyploid species contain heterogeneous ribosomes, with large subunits composed of 5S and 26S rRNA from different parental origins. Since the 5S rRNA is the only RNA type that binds ribosomal proteins before assembly into the ribosome (Ciganda & Williams, 2011), it would be intriguing to analyze ribosome proteins, the translatome, to explore potential variability in ribosomal proteins associated with the documented ribosomal RNA chimerism. Our findings suggest that heterogeneous ribosomes are fully functional, indicating that ribosome chimerism is tolerated by the cell and might even confer advantages. For instance, it is conceivable that heterogeneous ribosome could enhance proteosynthesis, potentially reflecting increased protein demands in allopolyploids.

### Chromosome rearrangements may alter expression of 5S and 35S rDNA in *Cardamine* allotetraploids

It is established that allopolyploids often undergo gene loss through diploidization, a process mediated by various mechanisms such as chromosome rearrangements, dysploidy, repeats elimination, transposition, and overall genome size reduction (Feliner et al., 2020; Weiss‐Schneeweiss et al., 2013). Consistent with the diploidization of *C. flexuosa* and *C. scutata*, we observed near‐complete (>99%) elimination of 35S rDNA from their H and P‐genomes, respectively. Therefore, the dominant expression of A‐genome 35S rRNA in *C. flexuosa* and *C. scutata* is attributed to the physical elimination of partner loci rather than epigenetic silencing. In contrast to 35S rRNA, the 5S rRNA genes appear to have longer retention time in *Cardamine* alloteraploids. For instance, *C. flexuosa* inherited nearly the additive number of *C. amara* and *C. hirsuta* genes (Table S8). Even in *C. scutata*, which lost the majority of the *C. amara* genes, the residual number still corresponds to about 100 copies. This aligns with previous observations in various systems, suggesting a greater stability of 5S loci compared to the 35S rDNA loci in allopolyploids (Alexandrov et al., 2021; Baum et al., 2008; Galián et al., 2014; Jang et al., 2016; Kellogg & Appels, 1995; Mahelka et al., 2013; Volkov et al., 2017). The loss of rDNA loci seems to have occurred without massive structural rearrangements of parental chromosomes, as indicated by the expected additive patterns observed with BAC probes in *C. flexuosa* (Mandáková et al., 2014; Figure S4).

While the mechanism of rDNA elimination remains unknown, it appears to be remarkably rapid, as observed in synthetic allopolyploids (Guo & Han, 2014; Pontes et al., 2004). In *C. flexuosa*, we previously identified an intergenomic reciprocal translocation, involving the translocation of 5S rDNA from the H‐genome to the A‐genome chromosome 4 (Mandáková et al., 2014). The recombinant chromosome carries both progenitor 5S rDNA arrays, which seem to form independent arrays, as suggested by cluster and FISH analyses. This translocation was consistently detected in all individuals collected from different populations, indicating its occurrence close to the allopolyploidization event (~10 000 years ago). Intergenomic translocations are relatively frequent in allopolyploids and may lead to deletions affecting plant phenotypes (Schilbert et al., 2023). For instance, in wheat‐rye hybrids, the translocation of rye 35S rDNA resulted in reduced copy number and loss of expression activity (Gustafson et al., 1988; Neves et al., 1995). Similarly, in the *A. thaliana* Lansbergis erecta ecotype, a partial translocation of active 5S rDNA from chromosome At5 to chromosome At3 led to the gain of heterochromatic marks and silencing (Simon et al., 2018). However, the identified translocation of 5S rDNA in *C. flexuosa* did not appear to affect the copy number or dominant expression of H‐genome 5S rDNA. Notably, the activity of the 5S locus was retained despite its close proximity to the breakpoint and a heterochromatic knob (Mandáková et al., 2014). Thus, intergenomic translocations do not uniformly alter expression patterns.

## CONCLUSIONS

In this study, we have provided, for the first time, evidence for uniparental silencing of 5S rDNA in plant allopolyploids, suggesting that nucleolar dominance (silencing of 35S rDNA) is accompanied by concurrent, but probably independent, silencing of 5S rDNA loci. Silencing and differential retention of progenitor rDNAs in allopolyploid genomes may result in the expression of rDNA variants from different subgenomes, leading to ribosome chimerism. We propose that epigenetic silencing and rDNA rearrangements contribute to another layer of variation in multi‐molecular ribosomal complexes that may contribute to the evolutionary success of allopolyploids.

## EXPERIMENTAL PROCEDURES

### Plant samples

We investigated the following *Cardamine* species: diploid *C. amara* L., *C. hirsuta* L., *C. parviflora* L. and *C. rivularis* Schur in Verh. (all 2*n* = 16), triploid *C*. × *insueta* (2*n* = 24), tetraploid *C. flexuosa* With. and *C. scutata* Thunb. (both 2*n* = 32). The examined accessions were previously characterized using comparative chromosome painting in our earlier studies (Mandáková et al., 2013, 2014, 2019). A list of the investigated accessions and their origins is provided in Table S1. Plants were grown from seeds and cultivated under standard conditions in growth chambers (150 μmol m^−2^ sec^−1^; 21/18°C, day/night; 16/8 h light/dark) or in a greenhouse (150 μmol m^−2^ s^−1^; 22/19°C, day/night; 16/8 h light/dark).

### Nucleic acids isolation and sequencing

Total RNA from fresh leaf tissue was isolated using a RNeasy kit (Qiagen, Hilden, Germany) following the protocol supplied by the manufacturer. DNA contamination was removed using DNase (Turbo DNA free; Ambion, Thermofisherscientific, Waltham, MA, USA). Total genomic DNA was isolated using a CTAB‐based method or with commercial kits such as Plant DNA mini (Macherey‐Nagel, Dűren, Germany). The concentration of nucleic acids was measured using a spectrophotometer (Implen N60, Implen GmbH, Műnchen, Germany), and quality was checked by agarose gel electrophoresis. High‐throughput sequencing including libraries preparations was carried out at commercial companies. A summary of the sequencing information and accession numbers of sequence reads archives within the PRJNA575831 Bioproject “Chromosome evolution in Cardamine hybrids and polyploid” (NCBI, GenBank, Rockville Pike, Bethesda, MD, USA) is available in Table S9.

### rDNA variation analyses

Raw RNA‐seq data (obtained from random primed or polyA libraries) or genomic reads were quality controlled using fastqc, trimmed with trimgalore/0.6.2 using default settings. Two million (1 million of pairs) of clean reads were used as an input for SNV calling. Out of these, 90–96% (Table S3) were mapped to the *C. amara* rDNA consensus 5S and 26S rDNA units. The consensus sequences were obtained from contigs after the cluster analysis (as below). The fidelity of reference sequences was verified by the alignment to the *A. thaliana* Columbia 5S rDNA sequence (GenBank AF198203.1). The mapping was carried out using commands in the CLC genomics workbench (Qiagen) (CLC) with the following parameters: Match score – 1, mismatch cost – 2, inser5tion cost – 3, deletion cost – 3, length fraction – 0.5 (26S rDNA)–0.9 (5S rDNA), and similarity fraction – 0.8 (i.e., >80% identity over the read length fraction). Read tracks were visually checked in the program window, and coverage graphs were constructed. Mapped reads files were used in SNV (i) and haplotype (ii) analyses: (i) Variants were called via the “Probabilistic Variant Detection” function tool in CLC using default settings. SNPs were filtered as follows: minimum read coverage – 400, count (the number of countable reads supporting the allele) – 40, frequency (the ratio of “the number of ‘countable’ reads supporting the allele” to “the number of ‘countable’ reads covering the position of the variant”): ≥5% (high‐frequency SNPs). In some polyA‐primed RNA‐seq libraries, the representation of 5S sequences was low. In these cases, the minimum read coverage parameter was reduced to –100 and minimum count – 10. (ii) To obtain 5S rRNA sequences, stand‐alone BLAST libraries were generated using a command in a CLC toolbox. Libraries were BLASTed against the reference 5S rDNA as a query. Reads were extracted from BLAST files using a command in CLC, trimmed keeping only long (>110 nt) sequences. If necessary, reads were subsampled randomly to about 200–600 reads/species for the phylogenetic analysis. Reads were renamed (Galaxy server tool), adding species‐specific codes. Multiple alignment containing sequences from a given allopolyploids plus reference sequence of each diploid progenitor was carried out, and a phylogenetic NJ tree was constructed. All calculations were run at default conditions using the Jukes‐Kantor model and 100 replicates. Reads from clades were extracted (command “Extract sequence list”), realigned and counted. Rare (<5 percent of input reads) highly mutated sequences falling out of the main clades were not counted. Variant counts and diversity analysis were estimated according to (Nei, 1987) (equations 8.4 and 8.12 but replacing 2n by n) computed by the DNaSp5 program (Rozas et al., 2003).

### Analysis of 5S rDNA arrays in long PacBio reads

We sequenced the *C. flexuosa* DNA (pop. Zelezne, SK) by the PacBio technology, yielding 33 154 reads for which a stand‐alone BLAST database was generated. The database was BLASTed against the 120‐bp *C. amara* 5S rDNA coding region. This resulted in multiple hits in 77 reads. Reads were then filtered: >90% identity; >2 kb lengths. This step reduced the number of reads to 22 reads. Reads were then BLASTed against the *C. flexuosa* 5S IGS major and minor family recovered from cluster 45 contigs in the RepeatExplorer analysis. Each of the 22 reads was subjected to self‐to‐self pairwise comparison by doplots.

### Estimation of rDNA genome proportion and copy number

The genome proportion and copy number of 5S and 26S rRNA genes were calculated from the number of mapped reads out of total reads according to procedures described by Wang et al. (2018). Briefly, the genome proportion of the rDNA was estimated from the ratio of the number of mapped reads divided by the total number of reads analyzed. The reference sequences were the 5S (120 bp) and the 26S rRNA gene (3391 bp) from the *Cardamine amara* consensus sequences obtained from mapped reads by a CLC command “Extract consensus sequence.” The single end (R1) Illumina reads were trimmed and used for mapping. Genome space occupied by the 5S/26S rRNA genes was calculated as Genome percentage (GP) of mapped reads × Genome size/100. Copy number was estimated by GP × GS/gene length, where GS is the genome size per haploid set. Genome size values were taken from the Plant C value database (https://cvalues.science.kew.org) (Bennett & Leitch, 2012) and from (Soga et al., 2021) (for *C. scutata*).

### Secondary structure modeling

Secondary structure modeling was carried out using an online tool at the RNAfold web server (Lorenz et al., 2011, http://rna.tbi.univie.ac.at/). The secondary structures were based on minimum free energy calculations using the Turner 2004 model. The program setting was as follows: isolated nucleotides were avoided; vote for dangling energies on both sides of a helix in any case. The eps format graphical outputs were exported to Adobe Photoshop for annotations.

### Whole‐genome cluster analysis

Genomic Illumina reads were sampled, renamed and trimmed in RepeatExplorer2 (Novak et al., 2013) at the Galaxy server. The raw 454 reads bearing large diversity of read lengths were trimmed to the length interval of 100–300 nt. A trigenomic comparative clustering was carried out according to the protocol described in (Garcia et al., 2023). Briefly, about 200 thousand genomic Illumina reads (pairs) from each allopolyploid and corresponding diploids were concatenated using text tools in a Galaxy server and subjected to clustering analysis using the RepeatExplorer2 pipeline. Clusters are by default identified by a BLAST threshold of 90% similarity across 55% of the read, with a minimum overlap of 55, 0.01% of cluster threshold and 40 as the minimum overlap for assembly. Clusters containing 5S sequences were searched using a query “5S” in a window. Low (<100 reads) abundant clusters were not considered. SeqGrapheR tool was used to visualize, explore and annotate the sequence‐based graphs generated by RepeatExplorer2. Clusters graphs were saved in a JPEG format and imported to Adobe Photoshop for further processing.

### Fluorescent *in situ* hybridization

Whole young inflorescences were fixed in freshly prepared ethanol:acetic acid (3:1) fixative overnight, transferred to 70% ethanol and stored at −20°C until further use. Chromosome spreads from fixed young flower buds containing immature anthers were prepared in accordance with established protocols (Mandáková & Lysak, 2023). Chromosome preparations underwent treatment with 100 μg ml^−1^ RNase in 2× sodium saline citrate (SSC; 20 × SSC: 3 m sodium chloride, 300 mm trisodium citrate, pH 7.0) for 60 min, followed by exposure to 0.1 mg ml^−1^ pepsin in 0.01 m HCl at 37°C for 5 min. Postfixation was then performed in 4% formaldehyde in 2× SSC for 10 min, with subsequent washing in 2× SSC twice for 5 min and dehydration in an ethanol series (70%, 90%, and 100%, 2 min each). The *A. thaliana* BAC clone T15P10 (AF167571), containing 35S rRNA genes, was utilized for *in situ* localization of nucleolar organizer regions (NORs), while the *A. thaliana* clone pCT4.2 (M65137), representing a 500 bp 5S rDNA repeat, served for the localization of 5S rDNA loci. All DNA probes were labeled with biotin‐dUTP, digoxigenin‐dUTP, or Cy3‐dUTP using nick translation, as outlined by Mandáková and Lysak (2023). Selected labeled probes were combined according to the experimental design and precipitated by the addition of 1/10 volume of 3 m sodium acetate (pH 5.2) and 2.5 volumes of ice‐cold 96% ethanol, followed by incubation at −20°C for 30 min. The resulting precipitate was then centrifuged at 13 000 ***g*** at 4°C for 30 min and resuspended in 20 μl of the hybridization mix (50% formamide and 10% dextran sulfate in 2 × SSC) per slide. Subsequently, 20 μl of the probe was pipetted onto a chromosome‐containing slide, with cover slips framed using rubber cement. The probe and chromosomes were denatured together on a hot plate at 80°C for 2 min and incubated in a moist chamber at 37°C overnight. Posthybridization washing was carried out in 20% formamide in 2 × SSC at 42°C. Immunodetection of hapten‐labeled probes followed the procedure detailed by Mandáková and Lysak (2023): biotin‐dUTP was detected using avidin–Texas Red (Vector Laboratories), amplified by goat anti‐avidin–biotin (Vector Laboratories), and further labeled with avidin–Texas Red; digoxigenin‐dUTP was detected using mouse anti‐digoxigenin (Jackson Immuno Research) and goat anti‐mouse–Alexa Fluor 488 (Invitrogen). Cy3‐dUTP‐labeled probes were observed directly. Following immunodetection, chromosomes were counterstained with 4′,6‐diamidino‐2‐phenylindole (2 μg ml^−1^) in Vectashield (Vector Laboratories).

### Southern blot hybridization

For Southern blots, the 5S rDNA probe was hybridized to the MboI‐restricted genomic DNA. The probe was ca. 472 bp insert of a clone carrying three copies of the 5S rRNA gene from *Artemisia tridentata* (GenBank; JX101915) (Garcia et al., 2012). The plasmid insert was amplified and labeled with the [32P]dCTP (DekaPrime kit; Fermentas, Lithuania). Hybridization was carried out at high stringency conditions (washing 2 × SSC, 0.1% SDS followed by 0.1 × SSC, 0.1% SDS at 65°C). The hybridization signals were visualized by Phosphor imaging (Typhoon 9410; GE Healthcare, PA, USA) and signals were quantified using ImageQuant software (GE Healthcare).

## AUTHOR CONTRIBUTIONS

The study was conceived by TM, MAL, and AK. TM and MAL collected/grew all plant accessions. TM, AK, RM, and AKr conducted high‐throughput sequencing. AK, TM, AKr, and RM carried out computing and bioinformatic analyses. Clustering analysis was done by AKr. Cytogenetic work was carried out by TM. AK and TM wrote the manuscript with inputs from ML, RM, and RV.

## CONFLICT OF INTEREST

The authors declared that they have no conflict of interest in this work.

## OPEN RESEARCH BADGES

This article has earned an Open Data badge for making publicly available the digitally‐shareable data necessary to reproduce the reported results. The data is available at https://www.ncbi.nlm.nih.gov/bioproject/PRJNA575831. All data from this work are found in a public GenBank repository within the SRA project PRJNA575831.

## Supporting information


**Figure S1.** A phylogenetic neighbor‐joining tree showing the composition of 5S rRNA types in diploid species: *Cardamine amara* (AMA), *C. hirsuta* (HIR), *C. parviflora* (PAR), and *C. rivularis* (RIV). Number of reads and a percentage are given for each species. The *C. amara*‐specific clade is circled.
**Figure S2.** Alignment of genomic 5S rDNA consensus sequences from *Cardamine* diploids. Proximal 5′‐prime and 3′‐prime and the genic (gray) regions are shown for each sequence. Dots are according to the *C. amara* sequence. Upstream and downstream regulatory elements are in orange boxes. Regulatory elements in the coding region are in brownish boxes. TIS, transcription initiation site.
**Figure S3.** Fluorescent *in situ* hybridization analysis of rDNA loci in 20 individuals of *Cardamine* × *insueta*. Metaphase chromosomes were subjected to hybridization using 5S (in red) and 18S (in green) rDNA probes. Chromosomes were counterstained with DAPI. Scale bars: 10 μm.
**Figure S4.** Fluorescent *in situ* hybridization analysis of rDNA loci in seven populations of *Cardamine flexuosa*. The 5S and 35S rDNA sites are represented in red and green, respectively. Each panel displays an upper row depicting metaphase stained with DAPI (left) and after hybridization with 5S (red), 35S (yellow) rDNA probes, and chromosome‐specific BAC clones (chr3 in green and chr7 in yellow). The bottom row of panels displays metaphase stained with DAPI (left) and after hybridization with 5S (red), 35S (yellow) rDNA probes, and chromosome‐specific BAC clones (chr4 in green). Ideograms illustrating hybridization patterns in karyotypes are presented on the right margin (upper panel, chr3 and chr8; bottom panel, chr4). BAC, bacterial artificial chromosome.
**Figure S5.** Detailed chromosome analysis showing the 5S rDNA locus in *Cardamine flexuosa*. Note a double signal on the chromosome bearing an intergenomic t(CA4) translocation.

**Table S1.** List of species used in this study, sequence archives, and type of analyses. Sheet 1: Sequence archives and type of analyses. Sheet 2: Accessions analyzed by FISH.

**Table S2.** 5S rDNA‐types and their frequency in RNA pools and genomic DNA. Sheet 1: Transcriptomic analysis. Sheet 2. Genomic analysis.

**Table S3.** Single nucleotide polymorphism analysis of 5S rDNA in populations of *Cardamine* diploids.

**Table S4.** Single nucleotide polymorphism analysis of 5S rDNA and 26S rDNA in *Cardamine* × *insueta*. Sheet 1: 5S rDNA analysis. Sheet 2: 26S rDNA analysis.

**Table S5.** Single nucleotide polymorphism analysis of 5S and 26S rDNA in *Cardamine flexuosa*. Sheet 1: 5S rDNA analysis. Sheet 2: 26S rDNA analysis.

**Table S6.** Single nucleotide polymorphism analysis of 5S and 26S rDNA in *Cardamine scutata*. Sheet 1: 5S rDNA analysis. Sheet 2: 26S rDNA analysis.

**Table S7.** Position of the 26S rDNA polymorphic sites in the diploid species.

**Table S8.** The 5S and 26S rDNA copy number in *Cardamine* diploid and allopolyploid species.

**Table S9.** The details of high‐throughput sequencing projects accomplished within this study.

## Data Availability

The raw genomic data of Illumina and PacBio genomic sequences, and transcriptome data have been deposited in the NCBI Sequence Read Archive within the Bioproject PRJNA575831. The biological materials, including those, will be shared by the contacts upon request.
