# Supplementary figures and images for: Uniparental silencing of 5S rRNA genes in plant allopolyploids – insights from Cardamine (Brassicaceae)
Source: Plant J. 2024 Jun 5;119(3):1313–26. doi: 10.1111/tpj.16850 (PMC13087485; doi:10.1111/tpj.16850)

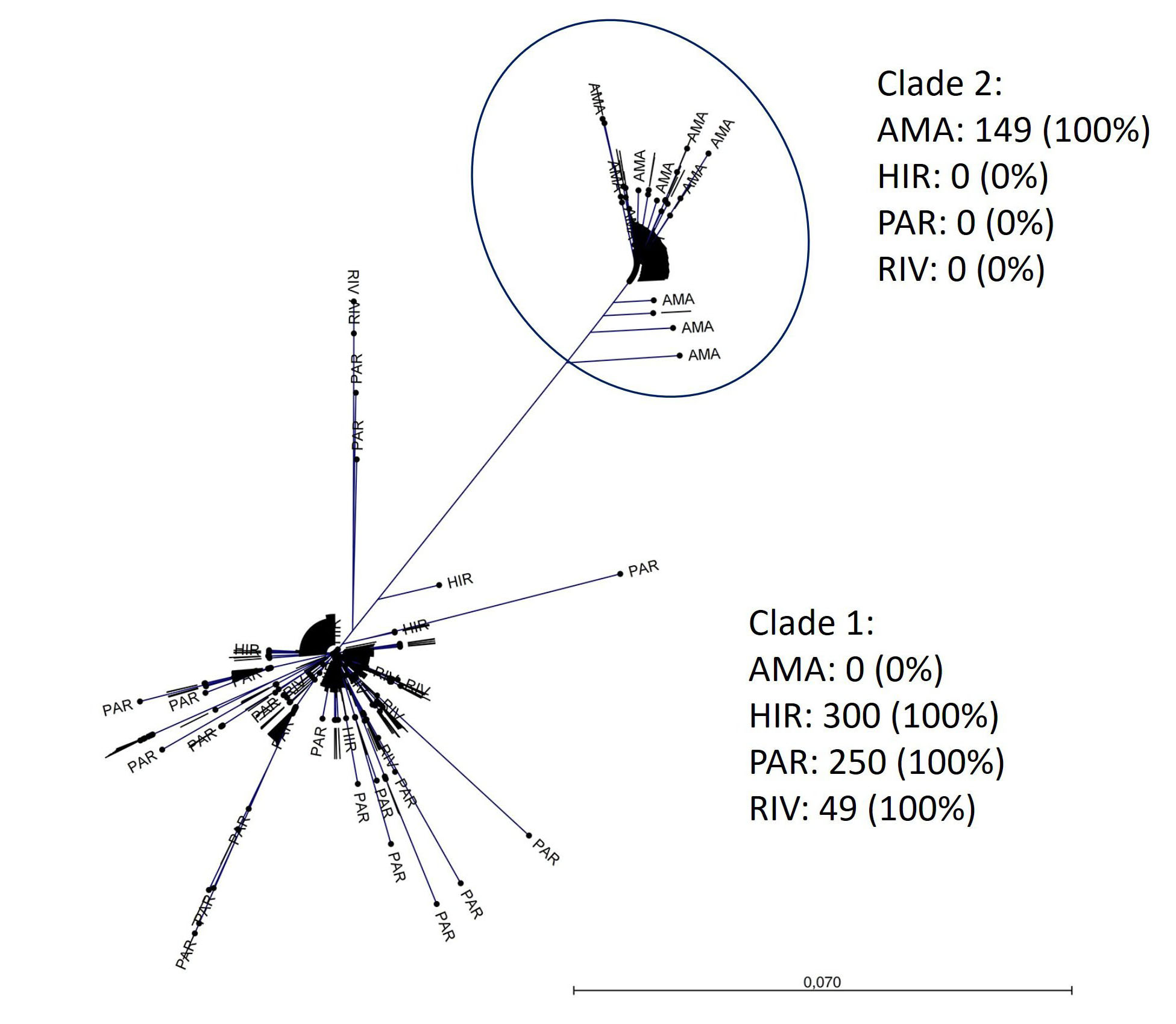

Supplement: Supplementary file 1 — Figure S1. A phylogenetic neighbor‐joining tree showing the composition of 5S rRNA types in diploid species: Cardamine amara (AMA), C. hirsuta (HIR), C. parviflora (PAR), and C. rivularis (RIV). Number of reads and a percentage are given for each species. The C. amara‐specific clade is circled. Figure S2. Alignment of genomic 5S rDNA consensus sequences from Cardamine diploids. Proximal 5′‐prime and 3′‐prime and the genic (gray) regions are shown for each sequence. Dots are according to the C. amara sequence. Upstream and downstream regulatory elements are in orange boxes. Regulatory elements in the coding region are in brownish boxes. TIS, transcription initiation site. Figure S3. Fluorescent in situ hybridization analysis of rDNA loci in 20 individuals of Cardamine × insueta. Metaphase chromosomes were subjected to hybridization using 5S (in red) and 18S (in green) rDNA probes. Chromosomes were counterstained with DAPI. Scale bars: 10 μm. Figure S4. Fluorescent in situ hybridization analysis of rDNA loci in seven populations of Cardamine flexuosa. The 5S and 35S rDNA sites are represented in red and green, respectively. Each panel displays an upper row depicting metaphase stained with DAPI (left) and after hybridization with 5S (red), 35S (yellow) rDNA probes, and chromosome‐specific BAC clones (chr3 in green and chr7 in yellow). The bottom row of panels displays metaphase stained with DAPI (left) and after hybridization with 5S (red), 35S (yellow) rDNA probes, and chromosome‐specific BAC clones (chr4 in green). Ideograms illustrating hybridization patterns in karyotypes are presented on the right margin (upper panel, chr3 and chr8; bottom panel, chr4). BAC, bacterial artificial chromosome. Figure S5. Detailed chromosome analysis showing the 5S rDNA locus in Cardamine flexuosa. Note a double signal on the chromosome bearing an intergenomic t(CA4) translocation. [file TPJ-119-1313-s008.zip › tpj16850-sup-0001-FigureS1.jpg]

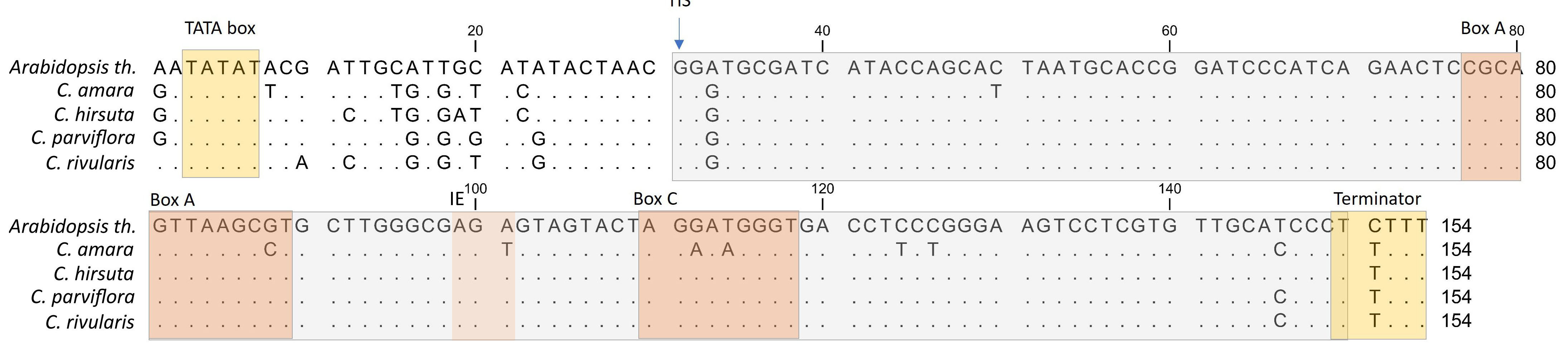

Supplement: Supplementary file 1 — Figure S1. A phylogenetic neighbor‐joining tree showing the composition of 5S rRNA types in diploid species: Cardamine amara (AMA), C. hirsuta (HIR), C. parviflora (PAR), and C. rivularis (RIV). Number of reads and a percentage are given for each species. The C. amara‐specific clade is circled. Figure S2. Alignment of genomic 5S rDNA consensus sequences from Cardamine diploids. Proximal 5′‐prime and 3′‐prime and the genic (gray) regions are shown for each sequence. Dots are according to the C. amara sequence. Upstream and downstream regulatory elements are in orange boxes. Regulatory elements in the coding region are in brownish boxes. TIS, transcription initiation site. Figure S3. Fluorescent in situ hybridization analysis of rDNA loci in 20 individuals of Cardamine × insueta. Metaphase chromosomes were subjected to hybridization using 5S (in red) and 18S (in green) rDNA probes. Chromosomes were counterstained with DAPI. Scale bars: 10 μm. Figure S4. Fluorescent in situ hybridization analysis of rDNA loci in seven populations of Cardamine flexuosa. The 5S and 35S rDNA sites are represented in red and green, respectively. Each panel displays an upper row depicting metaphase stained with DAPI (left) and after hybridization with 5S (red), 35S (yellow) rDNA probes, and chromosome‐specific BAC clones (chr3 in green and chr7 in yellow). The bottom row of panels displays metaphase stained with DAPI (left) and after hybridization with 5S (red), 35S (yellow) rDNA probes, and chromosome‐specific BAC clones (chr4 in green). Ideograms illustrating hybridization patterns in karyotypes are presented on the right margin (upper panel, chr3 and chr8; bottom panel, chr4). BAC, bacterial artificial chromosome. Figure S5. Detailed chromosome analysis showing the 5S rDNA locus in Cardamine flexuosa. Note a double signal on the chromosome bearing an intergenomic t(CA4) translocation. [file TPJ-119-1313-s008.zip › tpj16850-sup-0002-FigureS2.jpg]

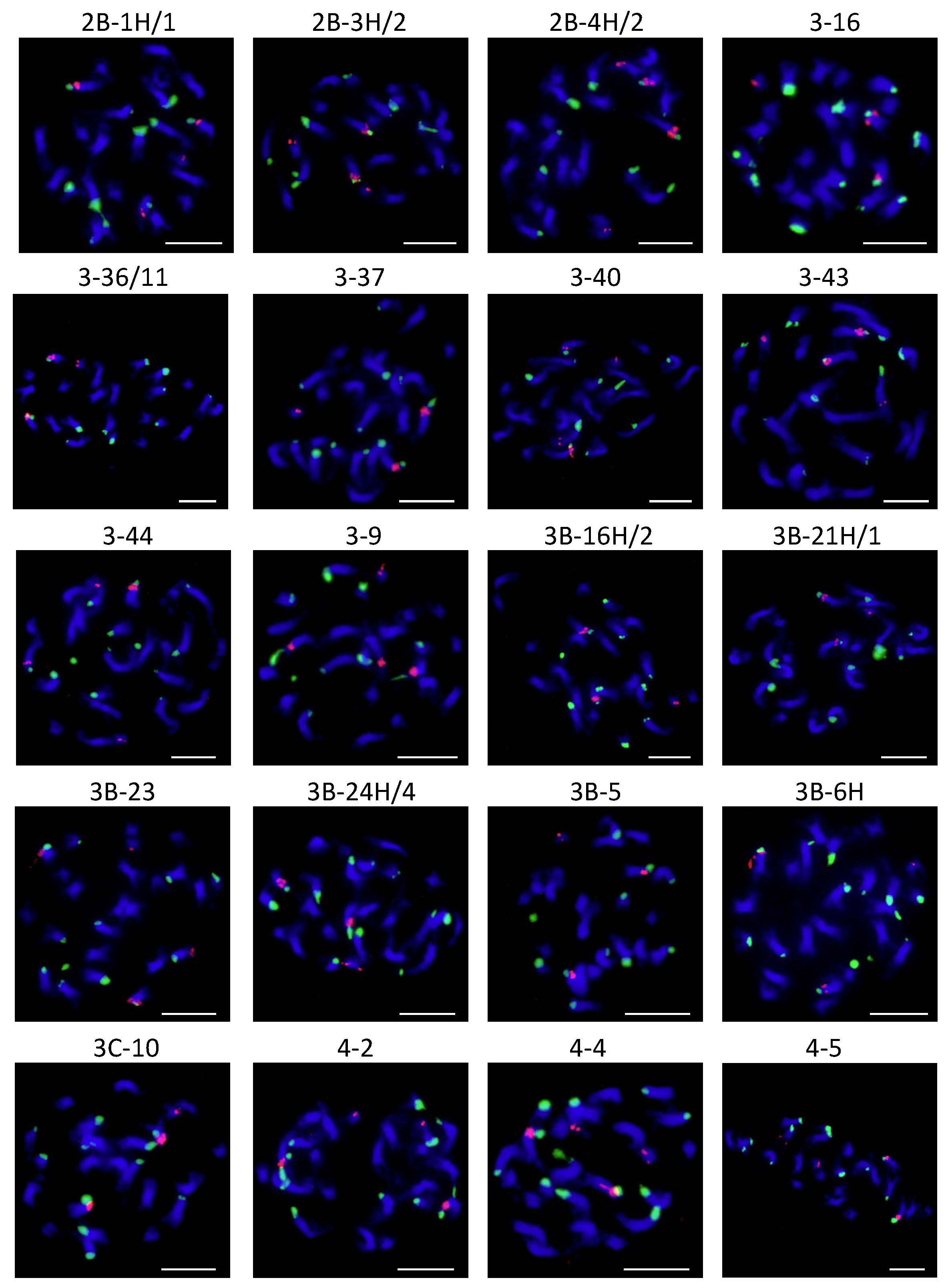

Supplement: Supplementary file 1 — Figure S1. A phylogenetic neighbor‐joining tree showing the composition of 5S rRNA types in diploid species: Cardamine amara (AMA), C. hirsuta (HIR), C. parviflora (PAR), and C. rivularis (RIV). Number of reads and a percentage are given for each species. The C. amara‐specific clade is circled. Figure S2. Alignment of genomic 5S rDNA consensus sequences from Cardamine diploids. Proximal 5′‐prime and 3′‐prime and the genic (gray) regions are shown for each sequence. Dots are according to the C. amara sequence. Upstream and downstream regulatory elements are in orange boxes. Regulatory elements in the coding region are in brownish boxes. TIS, transcription initiation site. Figure S3. Fluorescent in situ hybridization analysis of rDNA loci in 20 individuals of Cardamine × insueta. Metaphase chromosomes were subjected to hybridization using 5S (in red) and 18S (in green) rDNA probes. Chromosomes were counterstained with DAPI. Scale bars: 10 μm. Figure S4. Fluorescent in situ hybridization analysis of rDNA loci in seven populations of Cardamine flexuosa. The 5S and 35S rDNA sites are represented in red and green, respectively. Each panel displays an upper row depicting metaphase stained with DAPI (left) and after hybridization with 5S (red), 35S (yellow) rDNA probes, and chromosome‐specific BAC clones (chr3 in green and chr7 in yellow). The bottom row of panels displays metaphase stained with DAPI (left) and after hybridization with 5S (red), 35S (yellow) rDNA probes, and chromosome‐specific BAC clones (chr4 in green). Ideograms illustrating hybridization patterns in karyotypes are presented on the right margin (upper panel, chr3 and chr8; bottom panel, chr4). BAC, bacterial artificial chromosome. Figure S5. Detailed chromosome analysis showing the 5S rDNA locus in Cardamine flexuosa. Note a double signal on the chromosome bearing an intergenomic t(CA4) translocation. [file TPJ-119-1313-s008.zip › tpj16850-sup-0003-FigureS3.jpg]

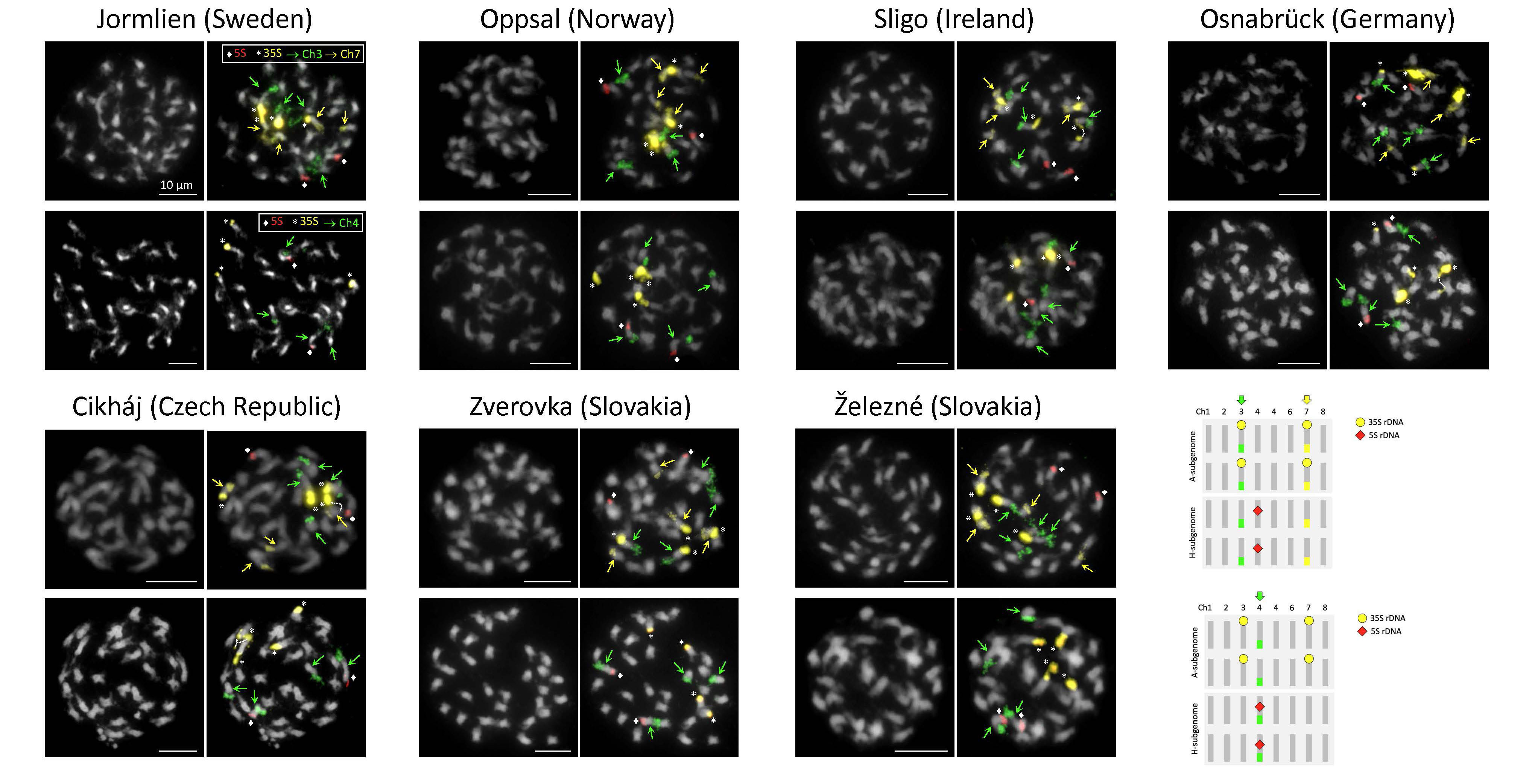

Supplement: Supplementary file 1 — Figure S1. A phylogenetic neighbor‐joining tree showing the composition of 5S rRNA types in diploid species: Cardamine amara (AMA), C. hirsuta (HIR), C. parviflora (PAR), and C. rivularis (RIV). Number of reads and a percentage are given for each species. The C. amara‐specific clade is circled. Figure S2. Alignment of genomic 5S rDNA consensus sequences from Cardamine diploids. Proximal 5′‐prime and 3′‐prime and the genic (gray) regions are shown for each sequence. Dots are according to the C. amara sequence. Upstream and downstream regulatory elements are in orange boxes. Regulatory elements in the coding region are in brownish boxes. TIS, transcription initiation site. Figure S3. Fluorescent in situ hybridization analysis of rDNA loci in 20 individuals of Cardamine × insueta. Metaphase chromosomes were subjected to hybridization using 5S (in red) and 18S (in green) rDNA probes. Chromosomes were counterstained with DAPI. Scale bars: 10 μm. Figure S4. Fluorescent in situ hybridization analysis of rDNA loci in seven populations of Cardamine flexuosa. The 5S and 35S rDNA sites are represented in red and green, respectively. Each panel displays an upper row depicting metaphase stained with DAPI (left) and after hybridization with 5S (red), 35S (yellow) rDNA probes, and chromosome‐specific BAC clones (chr3 in green and chr7 in yellow). The bottom row of panels displays metaphase stained with DAPI (left) and after hybridization with 5S (red), 35S (yellow) rDNA probes, and chromosome‐specific BAC clones (chr4 in green). Ideograms illustrating hybridization patterns in karyotypes are presented on the right margin (upper panel, chr3 and chr8; bottom panel, chr4). BAC, bacterial artificial chromosome. Figure S5. Detailed chromosome analysis showing the 5S rDNA locus in Cardamine flexuosa. Note a double signal on the chromosome bearing an intergenomic t(CA4) translocation. [file TPJ-119-1313-s008.zip › tpj16850-sup-0004-FigureS4.jpg]

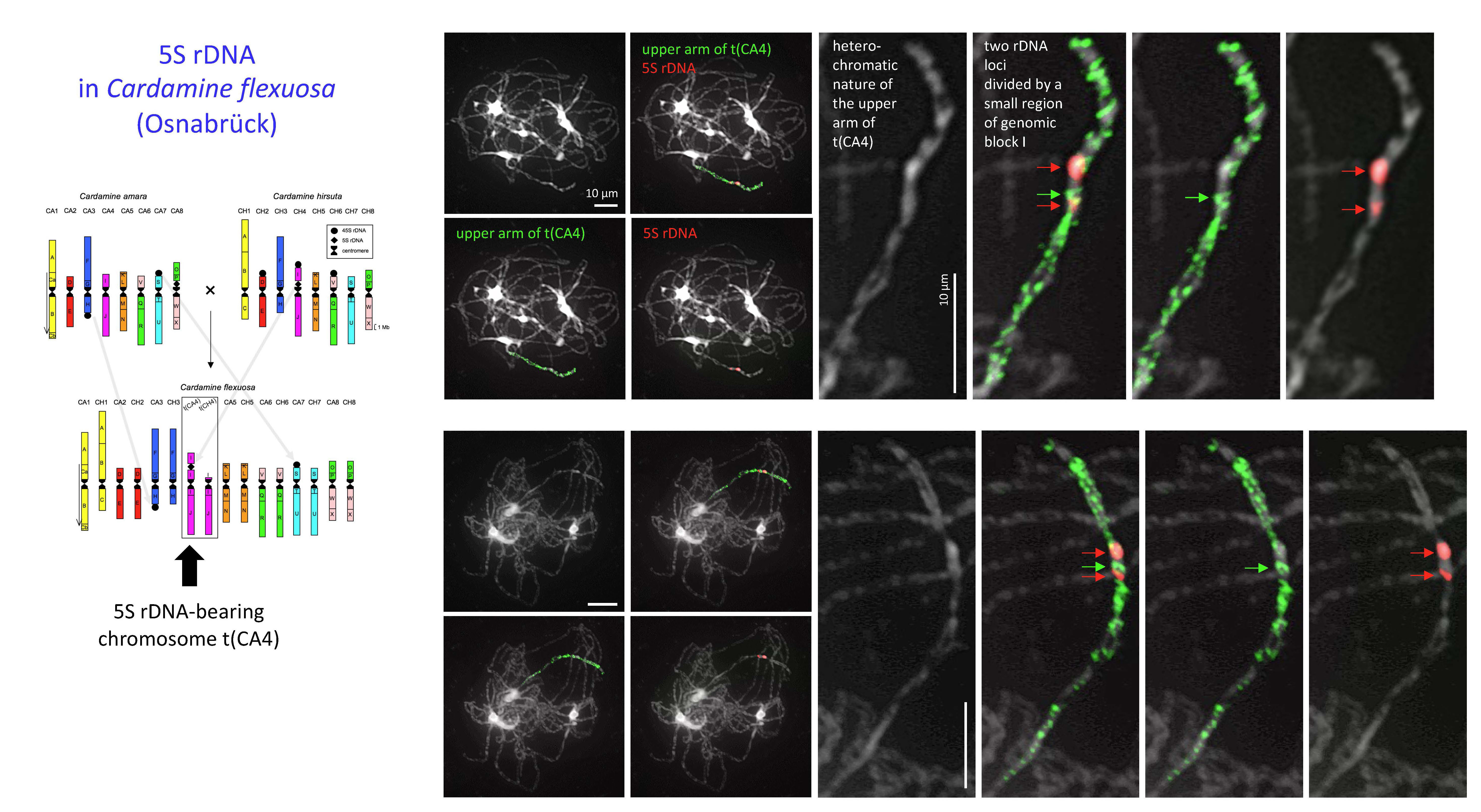

Supplement: Supplementary file 1 — Figure S1. A phylogenetic neighbor‐joining tree showing the composition of 5S rRNA types in diploid species: Cardamine amara (AMA), C. hirsuta (HIR), C. parviflora (PAR), and C. rivularis (RIV). Number of reads and a percentage are given for each species. The C. amara‐specific clade is circled. Figure S2. Alignment of genomic 5S rDNA consensus sequences from Cardamine diploids. Proximal 5′‐prime and 3′‐prime and the genic (gray) regions are shown for each sequence. Dots are according to the C. amara sequence. Upstream and downstream regulatory elements are in orange boxes. Regulatory elements in the coding region are in brownish boxes. TIS, transcription initiation site. Figure S3. Fluorescent in situ hybridization analysis of rDNA loci in 20 individuals of Cardamine × insueta. Metaphase chromosomes were subjected to hybridization using 5S (in red) and 18S (in green) rDNA probes. Chromosomes were counterstained with DAPI. Scale bars: 10 μm. Figure S4. Fluorescent in situ hybridization analysis of rDNA loci in seven populations of Cardamine flexuosa. The 5S and 35S rDNA sites are represented in red and green, respectively. Each panel displays an upper row depicting metaphase stained with DAPI (left) and after hybridization with 5S (red), 35S (yellow) rDNA probes, and chromosome‐specific BAC clones (chr3 in green and chr7 in yellow). The bottom row of panels displays metaphase stained with DAPI (left) and after hybridization with 5S (red), 35S (yellow) rDNA probes, and chromosome‐specific BAC clones (chr4 in green). Ideograms illustrating hybridization patterns in karyotypes are presented on the right margin (upper panel, chr3 and chr8; bottom panel, chr4). BAC, bacterial artificial chromosome. Figure S5. Detailed chromosome analysis showing the 5S rDNA locus in Cardamine flexuosa. Note a double signal on the chromosome bearing an intergenomic t(CA4) translocation. [file TPJ-119-1313-s008.zip › tpj16850-sup-0005-FigureS5.jpg]
